# Entrepreneurial Leadership and Entrepreneurial Success: The Role of Knowledge Management Processes and Knowledge Entrepreneurship

**DOI:** 10.3389/fpsyg.2022.829959

**Published:** 2022-03-29

**Authors:** Nida Hussain, Baoming Li

**Affiliations:** ^1^Business School, Zhengzhou University, Zhengzhou, China; ^2^Yunus Social Business Center, Zhengzhou University, Zhengzhou, China

**Keywords:** knowledge entrepreneurship, knowledge management processes, entrepreneurial leadership, entrepreneurial success, venture success

## Abstract

Various leadership styles have been widely studied to understand success. However, little research has empirically explored how entrepreneurial leadership (EL) influences entrepreneurial success (ES). Moreover, the role of knowledge management processes (KMPs) and knowledge entrepreneurship (KE) have been overlooked. Thus, using a knowledge-based view theory, this study aims to determine the relationship between EL and ES, mediated through KMPs. In addition, for a better understanding, the study also used KE as a moderator. A quantitative survey method has been employed to collect data from 390 entrepreneurial venture (EV) owners, co-founders, and managers in tech-oriented ventures (IT and Software-based) operating in Pakistan. Smart partial least squares (PLS) statistical software was used to analyze the impact mechanism of EL on ES through the structural equation model. The findings revealed that EL style positively influences ES. In addition, KMPs fully mediate the relationship between EL and ES. Furthermore, KE as a moderator, strengthens the relationships between EL and the knowledge management process. Theoretically, this study has complemented and enriched research on the influence mechanism between EL and ES. Practically, this study has important implications for leaders, managers, and founders to promote KMPs to achieve ES.

## Introduction

Leaders are usually known for their continual learning behavior that help them to sustain and achieve desired objectives in the form of success (Villaluz and Hechanova, 2019). Consequently, leadership styles vary from person to person. Entrepreneurial leadership (EL) is an association with passion for innovation, risk-taking ability, decision-making, and proactiveness (Gupta et al., 2004; Harrison et al., 2016; Al Mamun et al., 2018). The evolving EL style has extended its domain in firm enactment, venture success, and managerial development. EL has its role in refining organizational performance, which involves the pro-activeness, innovativeness, and risk-taking abilities of entrepreneurs (Dwi Widyani et al., 2020). It is pertinent to mention that EL is not a new paradigm (Huang et al., 2014; Leitch and Volery, 2017). The pivotal aspect of EL is to execute innovative ideas and business ventures (Leitch and Volery, 2017). In addition, the roadmap followed by EL is often full of uncertainties and risks, therefore, numerous strategies and approaches are adopted by EL to make their business successful (Hodgetts and Kuratko, 2004). Furthermore, EL provides opportunities to team members operating in different domains to foster entrepreneurial success (ES) (Huang et al., 2014; Alshanty and Emeagwali, 2019).

Entrepreneurial success has been acknowledged as a vital factor by entrepreneurs. ES is reported as a significant phenomenon for entrepreneurial perception (Fisher et al., 2014). Yet, this phenomenon of ES is frequently understood by the realization of a successful entrepreneurial venture (EV) (Staniewski and Awruk, 2019), and is sometimes associated with personal success. However, the research claims that success is further achieved through identifying leadership styles (Arham et al., 2017). Thus, a diverse leadership style contributes toward ES. Additionally, current theoretical and empirical studies reveal that a great variety of leadership styles influence successful business ventures and ES (Bertoldi, 2021). McKenzie and Sud (2008) discussed that the ultimate ES could learn from entrepreneurial failure. Baron and Henry (2011) suggested that ES cannot be calculated in monetary growth, it is associated with knowledge and implementation of different methods to attract investors toward business. Furthermore, success also counts as long term sustainability in the market. It is linked with other key drivers that help entrepreneurs step toward ES. Moreover, Visser et al. (2005) understand the relationship of transformational leadership through success. This study also stated that entrepreneurship could have a significant positive relationship with success. However, key functions of entrepreneurship as a subcategory of transformational leadership were still unclear. Entrepreneurial behavior also impacts the growth and success of an EV (Elia et al., 2020).

According to a research article published by Murphy (1992), technical training and workshops enhance the performance of leaders in terms of success. It has been suggested that leadership skill training plays an essential role in making an individual more capable, confident, and productive (IMD Leadership, 2021). On the other hand, entrepreneurs are risk-takers (Donbesuur et al., 2020), innovators (Thomas and Mueller, 2000), and competitors (Ali et al., 2020) in the comparative market. Therefore, such natural qualities and training polish EL hidden strengths that help them to achieve more success. However, leaders have persistent affection for knowledge management processes (KMPs) to venture execution and operations (Singh, 2008). Thus, in the context of a knowledge base view (KBV) theory by Grant (1996) mentioned that if any organization utilizes and implants the knowledge effectively and efficiently, it can lead, compete, take leverage in the competitive market. Shujahat et al. (2019) studied KBV and presumed that KMP (processes include knowledge conversion, acquisition, creation, protection, sharing, and utilization) plays a notable role in competitive advantage and improving performance. Another study shows that EL can be an essential and useful mechanism for knowledge sharing (KS) inside a business venture to take proper and rational decisions (Dirani et al., 2020). Numerous industries have recognized the implementation and incentive significance of KMP because the vitality of entrepreneurial orientation in a business venture depends on the amplitude of the implemented KMP (Latif et al., 2020).

García-Álvarez (2015) discussed a thorough strategy to make the right knowledge available at an accurate time. Hence, available and correct knowledge aids people to make precise decisions. Furthermore, accurate knowledge increases business competitiveness, which helps in ES. Abd Rahman et al. (2013) also argue about the response of KMP on the team skills and improvement of their business. Various research demonstrates that KMP plays a vital role in anticipation of individual capabilities toward the adoption of knowledge (Liao et al., 2007; Yeşil and Dereli, 2013).

Knowledge is considered a foremost manifestation of ES (Roxas et al., 2009). Knowledge entrepreneurship (KE) reflect innovation in any business (McDonald, 2002). Term KE is associated to identify the functions of entrepreneurship to the pipeline process in a proper way (Landström et al., 2012). According to McDonald (2002), it consists of four dimensions, including knowledge about environmental issues, conscientiousness in performing duties, a pledge to new projects, and acceptance of risky situations. However, with innovation, entrepreneurs mostly cooperate on these four dimensions. It is also stated that the ability of an organization to recognize new or existing knowledge as valuable, and as something to react to or exploit through the adoption of innovation (McDonald, 2002). Fernandes et al. (2017), reveals different factors as influential for KE, including detection of capabilities, entrepreneurial experience, and experience investing in other firms. Furthermore, the settings adopted by leadership are set to determine the general possibilities for KE to occur (Audretsch and Keilbach, 2007; Michelini, 2008). Thereby, the knowledge setting signifies the basic facts of the EV, such as its size, type of institution, business model, history, and historic approach to innovation. Under leadership, the style and values embraced by the current top decision makers, as well as the governance structure itself are evaluated (Cleveland and Cleveland, 2020; Bhatti et al., 2021). Thus, the concept of KE by a leader is central to the understanding of enabling or discouraging the conditions of business, as it adapts its attitude toward learning and whether values like innovativeness, competitiveness, and entrepreneurship, etc., are embraced or rejected (Leadbeater and Oakley, 2001; McDonald, 2002; Hayter, 2013).

Previous studies have only focused on EL styles influenced on the organization or firm performance (Nguyen et al., 2021), employee behavior (Bagheri, 2017), employee creativity (Newman et al., 2018), and workplace creativity (Cai et al., 2019). However, it was suggested that a relationship between EL and ES should be established (Viswanathan et al., 2014; Renko et al., 2015). It is noteworthy that limited literature is available in the domains of EL and ES. Thus, researchers need to pay more consideration to contribute in the area of EL in business studies (Al Mamun et al., 2018). Concerns have arisen about the relationship between EL and ES. Furthermore, knowledge management is considered one of the key drivers for ES (Gaimon and Bailey, 2013). Knowledge management is linked with intellectual capital and high tech EVs. Additionally, KS is considered a key element of success (Oliveira et al., 2020). However, a gap in KMPs needs to be addressed: mediation (Yao et al., 2020; Zahedi and Naghdi Khanachah, 2021). It has now been suggested that the role of KMP as a mediator should be validated, which could explicate the influence of leadership on distinct operational levels in business to achieve success (Soto-Acosta et al., 2018; Martins et al., 2019). Furthermore, the role of KE as a moderator has been rarely studied (McDonald, 2002; Kamal et al., 2020).

In recent years there has been considerable interest in leadership styles such as transformational leadership, transactional leadership, and paternalistic leadership style (Sorenson, 2000; Nogueira et al., 2018; Raziq et al., 2018), as a foundation of success. The relationship between leadership and success is studied, unlike with various mediating and moderating variables (Boga and Ensari, 2009; Elche et al., 2020; Muliati, 2020). This study identifies the knowledge gaps and employs them to contribute to literature and knowledge on EL by probing its implications for KMPs toward ES. Therefore, the following research is among the first to consider EL as a significant antecedent of ES. The research explores whether EL can act as an effective forecaster of KMPs. With this research, the quality of literature on KMP has been raised which is advantageous to recognize the role of KMPs in the progress of ES. Additionally, it subsidizes the literature related to leadership as it evaluated the novel practice of various leadership styles and KMPs, KE, and ES are rarely studied (Gupta et al., 2004; Renko et al., 2015; Dwivedi et al., 2020; Li et al., 2020). Hence, this research will deliver auxiliary perceptions to KBV theory by indicating how EL supports KMP toward ES and how EL support KMPs under the moderating effect of KE toward ES.

To the best of our knowledge, few research articles have explored the impact of EL on KMPs (Shamim et al., 2019). Hence under the consideration of KBV, the analysis will give insights into information by showing how EL supports KMPs that result in ES. The considering mechanism will assist to understand the EL effects on the ES. Moreover, the moderating role of KE will help to understand the relationship between EL and KMPs toward ES. KE is associated with up-to-date market trends. Such knowledge-based trends help entrepreneurs to evaluate their ventures in a more meaningful way. However, the KE environment is usually ignored by Pakistani entrepreneurs toward success. Hence, the following research focused on KBV (Cabrera-Suárez et al., 2001; Grant, 2013) to study the proposed research model under the context of Pakistani entrepreneurs. This research aimed to investigate the impact of EL on ES under the mediation of KMPs and moderation of KE on EL and KMPs toward ES in Pakistani on Tech-oriented business venture (Software/IT based companies). Based on the importance of EL, ES, KMPs, and KE, this study aims to identify the gaps in existing research. Therefore, the following research questions were proposed:

RQ1: Is there any direct impact of EL on ES in tech-oriented ventures in Pakistan?
RQ2: Do KMPs mediate the relationship between EL and ES in tech-oriented ventures in Pakistan?
RQ3: Does KE moderate the relationship between EL and KMP toward success in tech-oriented ventures in Pakistan?

The following paper is structured in six sections. Section “Literature Review” focuses on a detailed literature review. Section “Theory and Hypothesis Development” describes the theoretical framework and hypothesis development. Section “Methodology” defines the methodology, while section “Discussion” presents the results and discussion of the study. Finally, section “Conclusion” presents the conclusion, implications, and future research directions.

## Literature Review

### Entrepreneurial Leadership

Fløistad (1991) defined EL as a source of opportunity that revolves around achieving goals, creating chances in the job market and developing an environment for empowering people. EL can be differentiated among leadership and non-leadership skills, specifically entrepreneurial risk-taking behavior and innovative openness (Nicholas, 1998). Conversely, Gupta et al. (2004) refer to EL as “leadership that creates visionary scenarios that are used to assemble and mobilize a ‘supporting cast’ of participants who become committed by the vision to the discovery and exploitation of strategic value creation.” EL also creates the ability among individuals to identify the opportunities or market gaps, innovations, and solutions to comparative markets (Ireland et al., 2003). The vigorous visionary processes and creative features of entrepreneurship are discussed further in various studies (Kuratko, 2006). Furthermore, EL as a potential creator refers to a vision and mission that inspires and guides employees (followers) to make efforts and achieve set goals (Gupta et al., 2019). Therefore, EL requires determination to bring solutions for challenges, reduce uncertainty and risk in various stages of venture development.

Leaders are authorized to coordinate meritoriously inside an organization and solve challenging issues to improve and develop EVs (Aga et al., 2016). Different leadership styles are evident in entrepreneurial and transformational leadership (Pan et al., 2021). Transformational leaders focus on encouraging followers to achieve both corporate and personal goals (Zaman et al., 2020). Individualized attention is essential to transformational leadership, but it is not a component of EL. Contingent reward offers followers assistance in achieving self-actualization in transformational leadership (Al-Ghazali, 2020). Transformational and transactional leadership styles are portrayed as a person entrusted with an organizational task that must be completed within a certain organizational environment (Gupta et al., 2004). EL has to deal with new ideas and concepts which are not limited to an organizational level. Therefore, EL character should be more visionary, risk-taking, problem-solving, and involve strong decision making and strategic initiative (Renko et al., 2015). EL is perhaps not labeled as charismatic and inspirational as often as transformational leaders, even though they have principals with clear determination and objectives (Podsakoff et al., 1990). In addition, team-oriented leadership emphasizes efficient communication and collaboration, situational resolving, and interpersonal and group connections (Gupta et al., 2004). However, EL stresses route clearing for opportunity exploitation and value development.

Scholars have stated that leaders are supposed to be entrepreneurs (Czarniawska-Joerges and Wolff, 1991; Shane, 2010; Hubner, 2020). According to Henry et al. (2015), EL is a new level of leadership performance with high potential to bring novel change in the market. Hence, it enhances the performance of employees toward venture success and plays a vital role in any venture execution. EL provides a comparative advantage to innovative and newly discovered opportunities (Phangestu et al., 2020). EL assist individual to be successful leader, by solving critical problems and risk-taking abilities. Various arguments also lead that EL creates opportunities for materializing an innovative atmosphere for achieving goals (Si et al., 2015). EVs are developed with specialized leadership. Leaders undertake the development and maintain a profitable venture (Birley and Stockley, 2017). EL is a distinctive leadership style. Mainly concentrated on utilizing heterogeneous abilities to operate resourcefully and inventively in a competitive environment (Musa and Fontana, 2016). Gupta et al. (2004) outline that EL is acknowledged extensively around the globe and is most acceptable in the western world.

### Knowledge Management Processes

In the modern era knowledge is the most valuable asset for any organization. Primarily, Drucker (2012) specifies that raw material, products or services, available data or human minds are the basic source of knowledge industries. Nevertheless, these are the pivots for any organization or firm in order to perform tasks. Additional, knowledge creates market leverage with innovations and transforms (Maruf and Zhou, 2015). Jain (2007) intentionally recognized KMPs as an effective process of creating, storing, transforming, and sharing both learned and articulated knowledge to achieve required goals. Masa’deh et al. (2019) differentiated knowledge distribution outlining that it should be accurately divided and properly delivered to the right person at the right time to increase efficiency.

KM enabler permits leadership association with different organizations, to align KM behaviors with efficient strategies, policies, opportunities, effective communication, and enable learning processes (Yeh et al., 2006). Knowledge management endorses the values and offers metrics for determining knowledge influence in an organization. Consequently, leadership takes into account strategic challenges that help top level management consume available knowledge resources to upgrade competencies (Chin Wei et al., 2009). Moreover, acceptance of modern KS methods in an organization develops innovative and creative abilities in individuals. KMPs are a process by which a company creates, shares, uses, and manages knowledge. It is referred to a multidisciplinary approach that makes the best use of knowledge to achieve organizational goals. Hence, KMPs is an organizational learning facilitator (Jang et al., 2002; du Plessis, 2007).

According to Sadeghi and Rad (2018), knowledge acquisition (KA), KS, knowledge storage (KST), and knowledge applications (KAPP) are four major processes of KMP. KA refers to a process of retrieving, standardizing, and sorting information from a single source (Feroz et al., 2021). It practically initiates the venture to identify the market gap and opportunities to collect critical data from external extreme sources (Sousa and Rocha, 2019). KST is mentioned as a modern tool to store, sort, and organize available and collected data (Chou, 2005). KS confers the activity of sharing knowledge within an organization or customers according to their requirements (Wang and Noe, 2010). KAPP is defined as the effective and efficient use of available market, customer and competitor related data that helps in the achievement of desired objectives (Derek Ajesam et al., 2007). Utilizing KMPs accurately according to comparative knowledge provides an opportunity for organizations in which they can achieve their targeted desired goals and success (Obeidat et al., 2016). The pioneering work of Nonaka (1994) “*The knowledge-creating company*,” discusses how Japanese firms created innovation based on knowledge. The study uses Polanyi’s conception of “*tacit knowledge*” and cultivates a set of practical observations known as the SECI approach (*Socialization*, *Externalization*, *Combination*, and *Internationalization*). Under this approach knowledge is explicit and vice-versa (Nonaka et al., 1996, 2006; Durst and Runar Edvardsson, 2012).

### Knowledge Entrepreneurship

Knowledge entrepreneurship is a new concept. Initially, researchers define knowledge and entrepreneurship as individual entities. However, McKnight and Chervany (2001) state that an individual with the competence of knowledge and skill can implement various processes to execute a venture. KE refers to an ability that identifies an opportunity to comprehend an influential impactful product or service (Leadbeater and Oakley, 2001). KE differs from “traditional” entrepreneurial definitions, KE focuses on opportunities with improvement in knowledge creation and dissemination rather than monetary benefit (Ossai and Iwegbu, 2012). KE refers to aptitude by identifying a paradigm as an opportunity for taking action and intends to recognize innovative knowledge practice (Izzrech et al., 2013).

“Surfing the Long Wave: Knowledge Entrepreneurship in Britain” was a report that aimed to influence policy planning in the United Kingdom (Leadbeater and Oakley, 2001). This report was designated that “the entrepreneur is starting an enterprise that is based on knowledge work.” Though, this report was initial to start comments on KE. It was recommended by McDonald (2002) that KE is associated with innovation that leads any business, organization, or firm toward better performance: increasing KE mindsets increases innovation. Later, Coulson-Thomas (2003) discussed how knowledge-based opportunities (KBO) are diverse from resource-based opportunities (RBO). According to the author skill of acquiring, storing, sorting, and sharing exploited knowledge among team members is the first step toward success.

Skrzeszewski (2006) defines KE as an individual with skills and implementing skillsets on intellectual assets for new venture creations. With sufficient personal professional knowledge, an individual can generate value, profit, and opportunities. Furthermore, he argues that “The knowledge entrepreneur must know more about the subject at hand than his/her client or boss. It does not always have to be a great deal; sometimes the difference is based on the ability to communicate, present, or more importantly, apply the knowledge asset” (p. 3). However, Senges (2007) used McDonald’s (2002) model to propose the set of factors that directly shape the KE ability.

Researchers have also examined how entrepreneurial knowledge includes scanning, opportunity selection, strategy development, and association with management and leadership, which are interrelated tasks (Shane and Venkataraman, 2000; Chou, 2005; Singh, 2008; Unger et al., 2011). Anderson and Miller (2003) associated the different characteristics of human capital with entrepreneurial knowledge. They state that functional and theoretical knowledge are both important for understanding entrepreneurial awareness.

### Entrepreneurial Success

Entrepreneurial success is a complex phenomenon. Researchers argue that monetary or non-monetary factors can be a source of ES. Usually, ES is associated with venture success. Scholars have also stated that both entrepreneurial and venture success is the same (Bamford et al., 2004; Hogarth and Karelaia, 2012). In the end, entrepreneurs are focused on how they make themself and their venture successful. Some researchers also identified that gender inequality affects performance expectations and success. Men consider objective criteria to define success, such as obtaining prominence or acknowledgment of accomplishments, while women use intrinsic criteria to measure how they achieved their goals (Cliff, 1998; Burger-Helmchen, 2008). A metric of ES may help to identify actual and future successful ventures, as well as strengthen public policy aimed at increasing the success rate of a new venture (Fried and Tauer, 2009). Sometimes individuals also have strong willpower, which helps them to utilize resources effectively, reduce the extra cost to achieve success, and minimize entrepreneurial failure (Caliendo and Kritikos, 2008).

Entrepreneurial success is associated practically with economic or financial parameters (Zhou et al., 2019). Further, ES refers to strength and determination which endure the process of business execution and its remaining segments in the market for long run (Fisher et al., 2014). Only limiting ES with economic or financial indicators are not enough to understand the subjective criteria (Hogarth and Karelaia, 2012; Sarasvathy et al., 2013). Alstete (2008) shows that it i not necessary to associate ES with wealth but some associate it with work-life balance, this is mostly related to women entrepreneurs (Orlandi, 2017) case studies. Thus, for social entrepreneurship capital growth might not be a measure of success (Austin et al., 2006). However, substitute value creation and impactful activities are considered as supporting indicators to measure success (Thompson, 2004; Edelman et al., 2008). Therefore, ES is mostly associated with venture success.

When scholars argue about entrepreneurial opportunities, gaps or behavior toward success these arguments vary from researcher to researcher. Shane and Venkataraman (2000) suggested essential question for entrepreneurial researchers is “*Why, when, and how some people and not others discover and exploit opportunities*.” However, some argue about entrepreneurial behavior help entrepreneurs to grow and become successful (Mitchelmore and Rowley, 2010). EV success factors are associated with the opportunities availed by entrepreneurs (Renko et al., 2015). Entrepreneurship opportunity means a situation favorable for the success of EV. Every ES depends upon understanding the market situation, creativity, and growth opportunity. Moreover, leadership style holds a strong influence on success.

## Theory and Hypothesis Development

### Knowledge Base View

Knowledge base view identifies knowledge as a significant firm or business resource, it recognizes it as a strategic and vital resource to empower value creation, performance, growth, and success in business (Zack et al., 2009; Richey et al., 2010). Hence, strategic value creation can discover and utilized under the umbrella of EL (Gupta and Sharma, 2004). The operational implementation of KMP is to strengthen organizational learning skills. Learned skills use to promote personal experience and human resources at all levels. The certainty about the implementation of KMP diversifies and improves knowledgeable capital (Ramadan et al., 2017). Consequently, a successful business understands that they should directly attend the KMP to develop, create, spread, and continue knowledge (Masa’deh et al., 2019).

Knowledge base view researchers consider firms should substitute practices for successful apprehension, including the collection, evaluation, distribution, and publication of knowledge apprehended inside their operational activities. Firms can adapt and develop innovative processes, tactics, and strategies for connecting with their team members to gather their data for future insights. Nickerson and Zenger (2004) distinguish that a primary task of management is to “sustain above-average profits by continually discovering new knowledge or new solutions that form unique combinations on existing knowledge.” Although some researchers argue that knowledge contributes to the growth of a business’s technical competencies and these activities enable employees to share cross knowledge (Szulanski, 1996).

Under the light of KBV (Grant, 1996), to achieve better performance, effective and efficient deployment of KMP is important as it will lead to the successful utilization of knowledge-based resources (Mahdavi Mazdeh and Hesamamiri, 2014). According to KBV, specific capabilities and performance increase when knowledge is managed effectively (Leal-Rodríguez et al., 2013). KBV agrees that knowledge is generated, stored, and exploited by entities with coordination and integration organizational requirements, not by a single entity (Miles, 2012).

### Relationship Between Entrepreneurial Leadership and Entrepreneurial Success

Bass (1985) studied how leadership styles usually impact the level of performance and augmentation in any organization. Entrepreneurs successively execute their businesses with strong leadership commitment, which help them to maintain a successful project for the future. Bhattacharyya (2006), also argue that “successful business executives are not only good leaders but invariably turn out to be good entrepreneurs as well.” Therefore, a leadership style that generates a suitable comparative environment for entrepreneurship and innovation in the market leads to success.

Some preliminary work was carried out in the 1990s, which drew the attention of numerous researchers to highlight entrepreneurial activities as a driving force for economic growth and development (Wennekers and Thurik, 1999; Peris Bonet et al., 2011). Researchers believe that ES is associated with stable economies (Shakeel et al., 2020; Urbano et al., 2020). Nonetheless, ES is a complex phenomenon which usually associates with numerous measurements (Dej et al., 2013). Various parameters have their influence on success. Kim and Hann (2019) specified that success is not necessary for every business launched in the market. He also assumed that the assertiveness of entrepreneurs encourages the extension of the business. Hence a business success can be determined over the business momentary performance more specific to generating profits, investments, and productivity.

Entrepreneurship has increasingly been known as the well-admired pathway to performance and market renewal (Viswanathan et al., 2014). This suggests that opportunity is required to develop the relationship between EL and ES. EL can provide a platform to teams in such an environment where they can collect, share, and utilize knowledge effectively and proposed possible solutions (Renko et al., 2015). Many experts now believe that process-oriented culture in business tightly control organizational administration (Ubaid and Dweiri, 2020) enhances performance (Upadhyay and Kumar, 2020), which later leads to success. The above-mentioned features are the key elements of EL that influence ES. Thus, keeping context in mind, the following hypothesis is proposed:

H1. *Entrepreneurial leadership has a positive influence on entrepreneurial success*.

### Relationship Between Entrepreneurial Leadership and Knowledge Management Processes

Knowledge adoption and skill learning are categorized processes of learning, which hold a significant association with leadership development and behavior (Vera and Crossan, 2004). This underlines that impact of learning directly influences the strategic position of a leader to interact with lower and middle levels of management (Jyoti and Dev, 2015). Hence, without learning it is not possible to achieve goals. In addition, Coulson-Thomas (2004) argue that in the past, training, and development were not considered significant sources of incremental profit, rather than engaging in income generation, the focus was predominantly on cutting costs. However, real-time training, skill development, and mentoring are cost efficient in the long run (Sullivan, 2000). Recently, an increasing number of studies have found that basic education, professional training, skills based training, and professional experience have a significant impact on ES (Kurczewska et al., 2020). Likewise, some researchers contend that education is an extrinsic element that is assumed to be one success factor (Kolstad and Wiig, 2013; Ferreira, 2020; Van der Lingen et al., 2020).

It is important to provide an educational platform for EVs. When entrepreneurs participate in educational and business incubation their learning and market skills are upgraded (Shepard, 2013). Organizations are prevailed on to formulate various training modules that are contingent upon the requirements of distinct employees (Abd Rahman et al., 2013). The focus is to buoy up the trained employees to implement their skills (knowledge) and develop a knowledge based atmosphere that will improve policies to preserve these employees (Alagaraja et al., 2015). In this regard, the Government of Pakistan provides various training facilities for entrepreneurs (SMEDA, 2021). There are various platforms such as the Small Medium Enterprise Development Authority (SMEDA), National Incubation Centers (NIC), Chamber of Commerce, and other governing bodies that are directly and indirectly associated with entrepreneurs in order to guide Pakistan according to market demand (Iqbal and Malik, 2019).

The positive intentions of leaders toward knowledge flow, inside any organization, significantly encourage their team members to think innovatively (Rupčić, 2020). In contrast, poor communication and lack of knowledge could damage innovation, and does not motivate employees to absorb new information (Lam et al., 2021). A study by Knockaert et al. (2011) identifies the significance of knowledge transfer as a means of enhancing performance in technology based organizations from top level management to team members. Based on the above discussion, we propose the following hypothesis:

H2. *Entrepreneurial leadership has a positive influence on knowledge management processes*.

### Relationship Between Knowledge Management Processes and Entrepreneurial Success

For any business to achieve success, KMPs are considered important for growth in terms of its intellectual capital (Hussinki et al., 2017). All the aspects of KMP hold a strong influence by the intellectual capital and employee knowledge (Seleim and Khalil, 2011). Most KMPs target to apprehend, acquit, authenticate, and share knowledge. Mehralian et al. (2014) indicate that knowledge acquirement in any venture shows the capability to regulate, establish, and achieve information from peripheral resources and its dynamic toward success. Therefore, the modernization and novelty of existing knowledge reveal the precarious role of KMPs in the improvement of human resources and achieving success.

Venture success and growth is usually associated with entrepreneurs (Zorn and Taylor, 2004). This is also supported by other scholars who recognize that decisions to grow in the market are made by the entrepreneur themselves (Baumann-Pauly et al., 2016), thus for some entrepreneurs most important characteristic is innovativeness (Drucker, 2014). Entrepreneurs cannot only depend on decision making as knowledge and skills are the most important pillars of growth and success (Kor and Mahoney, 2005; Mazzarol and Reboud, 2006). Thus, we assumed:

H3. *Knowledge management processes has a positive influence on entrepreneurial success*.

### The Mediating Role of Knowledge Management Process Between Entrepreneurial Leadership and Entrepreneurial Success

Leaders play a decisive role in the processes of management in information systems. At a certain level, leaders are visionaries, motivators, processors, and provide frameworks that enhance learning capabilities (Bryant, 2003). Some studies have shown the importance of leadership in KMPs. Tanriverdi and Venkatraman (2005) discovered that the proliferation of information sharing is dependent on a firm’s technical capability. Bavik et al. (2018) had a detailed look at the ethical leadership relationship with KMPs and discovered that efficient leadership styles affect the operational process. Hence, EL prefers to improve a person’s knowledge, abilities, and competencies (Leitch and Volery, 2017); they react and transform by augmentation of present knowledge and expertise (Durst and Runar Edvardsson, 2012; Huang et al., 2014). With knowledge implementation, a shift in focus has occurred from technological advancements (Nadolska and Barkema, 2007; van der Westhuizen and Goyayi, 2020). Strategies are designed to move inputs and products to information and knowledge, altering organizations and considering the basis for competition (Dhir et al., 2020).

Organizations are adapting to a changing external environment, which puts high demands on leaders to provide different skills, knowledge, and practices (Jansen et al., 2009). KMPs are rapidly adopted by organizations to sustain their growth. In most cases, success is associated with KMPs (Liebowitz, 1999; Gray, 2006; Paramsothy et al., 2013). The performance of any business can be improved by using KM, to sustain its competitive advantage through the accomplishment of targeted work and goals (Zorn and Taylor, 2004; Jaleel et al., 2019). Taking advantage of knowledge is critical for any organization. For ES, it is noteworthy to manage knowledge effectively. Hence, the critical role of KM in KS and acquisition play vital role in success (Headd, 2003). For organizational performance, KM aims to create and acquire credible use of knowledge that allow employees easy access to data usage (do Adro and Leitão, 2020). Firms use KM to gather and create potentially useful information and make it accessible to their employees and customers to ensure organizational development and performance (Aliyu et al., 2015).

Employees’ attitudes toward conducting knowledge tasks and participating in the KMPs are forged by transformational leaders who often create a knowledge supporting culture in the form of establishing a collection of values, assumptions, and beliefs relevant to knowledge (Birasnav et al., 2011). Implementation of this culture holds influence on success. Moreover, leadership, according to Wei and Miraglia (2017), is a KM enabler. According to his study within the organization, KM enables the support of KM performances with opportunities, structural policies, interconnecting the best strategies, endorsing the values of KM, and providing indicators for measuring information effects. Therefore, leadership has a substantial influence on KS among team members. Zhu et al. (2018) discuss the financial and non-financial incentives shared through KS help organizations to develop new products and suggest cost-effective methods. Thus, it is challenging to attain KM success without the dedication of leadership (Civi, 2000). Thus, keeping context in mind, the following hypothesis is proposed:

H4. *Knowledge management processes mediates the relationship between entrepreneurial leadership and entrepreneurial success*.

### Moderating Role of Knowledge Entrepreneurship

Under the shadow of entrepreneurial opportunities, knowledge base inventions are characterized as scientific and non-scientific (Drucker, 2014). Knowledge fascinates all entrepreneurial sources to gain success and financial benefits. However, to succeed, knowledge base innovation would demand all aspects of knowledge related to innovation, entrepreneurship, and knowledge itself. KE comes from education, skill, and experience (Donnellon et al., 2014). The entrepreneur should understand the balance of available knowledge of all domains rather than only focusing on specific knowledge domains (Iversen et al., 2009). Argyris and Ransbotham (2016), observed and proposed, a new prototype of project leadership called “Knowledge Entrepreneurship” that incorporates KMP in the domain of managerial skills and technological adaptability. Scholars work on socio-economic institutional complex networks to develop new shapes of latest technology, which effect knowledge entrepreneurs (Chandler, 1990; Garud and Karnøe, 2003; Christensen, 2004).

Knowledge is one of the most significant predictors of ES. According to Makhbul and Hasun (2011), sources of knowledge vary from personal experience to private and formal/informal education. He mentioned that a well-informed (educated or aware) entrepreneur can pioneer and elicit innovative ideas, which empower entrepreneurs to grab opportunities evolving from the market.

There is massive potential for KE toward improving performance and attracting customers and stakeholders (Coulson-Thomas, 2003) based on knowledge. KE use the “know-how” for their demand and make their competencies commercial to craft idiosyncratic assistance and arrange for customers with modern incentives (Coulson-Thomas, 2003, 2012). Based on the above arguments, the following hypothesis was developed:

H5. *Knowledge entrepreneurship moderates the positive relationship between entrepreneurial leadership and knowledge management processes in the way that the relationship will be stronger when there is high knowledge entrepreneurship*.

**FIGURE 1:**
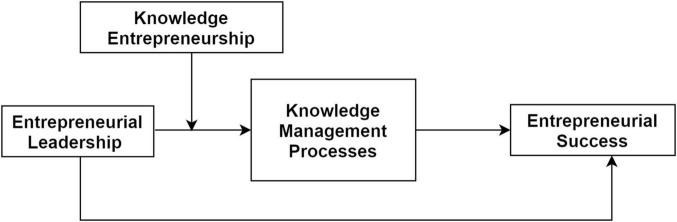
Conceptual model.

Based on KBV theory and the proposed hypothesis, a conceptual model (see Figure 1) has been developed to understand the relationship between variables.

## Methodology

### Sample and Data Collection

A quantitative approach was used in this study. The participants were from various cities in Pakistan. Using convenience sampling, data were collected from founders, co-founders, and entrepreneurial leaders of tech-oriented ventures (Bagheri et al., 2020) including IT and software-based companies (Donate and Sánchez de Pablo, 2015). Because KM strategies at IT companies tend to be broader, the management of knowledge is emphasized more in their processes. In addition, technology-intensive industries are based on knowledge that requires a different managerial approach than those in non-knowledge industries. Therefore, in certain situations leadership and HR management play a unique and supportive role in cultivating and implementing KMPs (Yahya and Goh, 2002).

The participant’s collaboration in the research was primarily elicited through telephone. Consequently, after pre-test and alteration, the self-administered structured questionnaire and its cover letter were emailed to each participant. Through email, we also inform each venture owner about the significance of the study and highlight the importance of their feedback by filling out questionnaires. For the follow up at 25 days, a reminder email was sent to non-responding participants. In addition, we also requested co-founders and entrepreneurial leaders to relay the email to other fellow co-founders and entrepreneurial leaders who also belonged to the target population of interest (Nunan et al., 2020).

A total of 430 questionnaires were distributed. Conversely, a total of 390 completed questionnaires were received. The total response rate was 65.69%. Furthermore, the collected data shows the demographic description that male participants were higher in number. The average age of respondents was between 31 and 40 years. The majority of participants had bachelor’s degrees, worked as IT/Software engineers, and had experience of between 1 and 5 years. Related details are mentioned in Table 1.

**TABLE 1:** Participant demographics description.

Demographics category	Frequency	Percentage
**Gender profile**		
Female	189	48
Male	201	52
**Age profile**		
18–20 years	52	13
21–30 years	108	28
31–40 years	145	37
41 years above	85	22
**Education profile**		
High school	26	7
Intermediate	86	22
Bachelor	188	48
Master	76	19
Above masters studies	14	7
**Working experience**		
1–5 years	241	62
6–10 years	92	23
Above 10 years	57	15
**Working domain**		
IT/software engineers	129	33
Gaming developer	45	11
Android developer	98	25
Website developer	62	16
Education	22	6
Other	34	9

Following the recommendations of Armstrong and Overton (1977), we made certain that non-responding bias was not a major concern and independent samples *t*-test was executed. We divided respondents into two sets; the first follow-up (early responders) based on participants who initially responded to the questionnaire and the second follow-up (late responders) who answer back after 25 days of reminder. Those who replied after the second follow-up are recorded as comparable to non-respondents (Armstrong and Overton, 1977). Hence, an independent samples *t*-test was executed to ensure that no significant difference was found among early and late respondent EL, KMP, KE, and ES. In the present study, non-responding bias was not a foremost issue. Additionally, self-reporting scales were exploited in this study, to confirm that common method bias was implemented by Harman’s single factor test (Podsakoff and Organ, 1986). Hence, common method bias was not a serious issue in the present study.

### Measures

A 5-point Likert scale was used in a questionnaire that mentioned “1” to “strongly disagree” to “5” to “strongly agree.” Items were taken from previous existing studies after understanding the variable of interest.

#### Entrepreneurial Leadership

The study adopted an 8-item scale (Cronbach’s alpha = 0.89) developed by Renko et al. (2015). The items in this scale reflect the leadership styles and their impact on success. EL construct includes: “*I have creative solutions to problems, and challenges push me to act more innovatively*.”

#### Knowledge Management Process

Based on the four constructs of KMPs (KA, KS, KST, and KAPP); KMP was measured using a 44-item scale developed by Gold et al. (2001). The KMP comprised of 44-items including 8-items on KA, 10-items on KU, and 10-items on KP (Cronbach’s alpha = 0.85). KS used a 10-items scale (Cronbach’s alpha = 0.94), adopted from Donate and Guadamillas (2010). KMP items include “*In our organization, the organizational procedures are documented through work procedures, written protocols, handbooks, etc.*,” “*Has processes for acquiring knowledge about new products/services within our industry*,” “*Has processes for converting competitive intelligence into plans of action*,” and “*Can locate and apply knowledge to changing competitive conditions*.”

#### Knowledge Entrepreneurship

The scale of KE was adopted from McDonald (2002), 5-items (Cronbach’s alpha = 0.85). KE items included “*We systematically process and analyze information about competitors?*”

#### Entrepreneurial Success

Using Fisher et al. (2014), 9-item (Cronbach’s alpha = 0.71) scale to measure ES based on individual and venture success. The ES measured include: “*exceed the business goals I set out to achieve in founding at least one business and build a business sustainable beyond my involvement*.”

### Data Analysis and Results

The data harvested from the questionnaire survey were analyzed through the partial least squares structural equation modeling (PLS–SEM) with the path modeling method. The motivation for selecting PLS path modeling was its widespread acceptance in disciplines of management sciences (Hair et al., 2012; Sarstedt et al., 2014a). Furthermore, the present study aimed to predict the dependent variable (ES) and it is known as the “most fully developed and general system” in SEM studies (Moustakas, 1994). Two-step approaches include the Measurement model and the Structure model was used in the following study.

#### Measurement Model

The measurement model is also known as the “Outer Model.” It exhibits the relationship between constructs and indictors. It is consist of composite reliability (CR) to calculate internal consistency, individual indicator reliability, and average variance extracted (AVE) to assess convergent validity (Hair et al., 2012; Sarstedt et al., 2014a). It is used to evaluate the acceptability of the scales used.

In individual item reliability, each item is evaluated based on its outer loadings (Duarte et al., 2010; Sarstedt et al., 2014b). Researchers consulted a rule of thumb by recommending items whose density is between 0.40 and 0.70 (Hair et al., 2014). However, constructs value lower than 0.6 should be removed (Gefen and Straub, 2005). Hence, the present study was satisfactorily above or equal to the value of 0.6 and more because outer loadings for respective latent variables meet the item reliability criteria (refer to Table 2). Consequently, this study meets the reliability criterion successfully.

**TABLE 2:** Factor loading values with Cronbach’s alpha, CR, and AVE.

Latent variable	Construct	Loadings	Cronbach’s alpha	Composite reliability (CR)	Average variance extracted (AVE)
Entrepreneurial leadership	EL_1	0.763	0.78	0.848	0.53
	EL_2	0.773			
	EL_3	0.754			
	EL_4	0.727			
	EL_5	0.611			
Entrepreneurial success	ES_1	0.727	0.794	0.866	0.618
	ES_2	0.801			
	ES_3	0.843			
	ES_4	0.769			
Knowledge application	KAPP_1	0.773	0.739	0.834	0.557
	KAPP_2	0.732			
	KAPP_4	0.687			
	KAPP_5	0.789			
Knowledge acquisition	KA_1	0.732	0.769	0.852	0.591
	KA_2	0.802			
	KA_3	0.789			
	KA_4	0.75			
Knowledge storage	KST_1	0.743	0.862	0.891	0.66
	KST_2	0.748			
	KST_3	0.654			
	KST_4	0.726			
	KST_5	0.774			
	KST_6	0.715			
	KST_7	0.775			
Knowledge sharing	KS_1	0.712	0.862	0.891	0.54
	KS_2	0.731			
	KS_3	0.894			
	KS_4	0.893			
Knowledge entrepreneurship	KE_1	0.821	0.845	0.89	0.619
	KE_2	0.825			
	KE_3	0.824			
	KE_4	0.729			
	KE_5	0.726			

In CR, researchers proposed an approximated value that should be greater or equal to 0.7 for the consideration of coefficient (Hair et al., 2014). This study demonstrates the CR coefficients for each of the latent variables ranged above 0.75. Therefore, this study signifies the sufficient internal CR of the measures (Hair et al., 2011).

The valuation of convergent validity with AVE has been endorsed by Fornell and Larcker (1981). Nevertheless, Chin (1998) recommended that for any specific construct the value of AVE should be less than 0.50 and more to designate the convergent validity. The AVE values mentioned in Table 2 directed that the AVE value has been attained at least possible of 0.50; consequently (Chin, 1998), it is determined that the present study established passable convergent validity (Chin, 1998). In addition, Hair et al. (2011) stated same that the estimated value of loading factors in the measurement model should be above 0.6 and AVE values should be above 0.50. Likewise, the CR should be greater than AVE (Hair et al., 2012). Table 2 shows the values of loadings, Cronbach’s alpha, CR, and AVE, which supports the convergent validity of the proposed model.

#### Discriminant Validity

Discriminant Validity of variable identifies the level up to which constructs correlate and indicators signify only a particular construct (Hair et al., 2012). The present study followed the heterotrait–monotrait (HTMT) ratio procedure for the discriminant validity of the constructs. According to this technique threshold values of the HTMT ratio should equal or 0.90 (Henseler et al., 2016). Thus Table 3 fulfils HTMT ratio procedure.

**TABLE 3:** Discriminant validity following Fornell and Larcker criteria.

	EL	ES	KA	KAPP	KS	KST	KE
EL	**0.728**						
ES	0.087	**0.786**					
KA	0.483	0.059	**0.769**				
KAPP	0.519	0.075	0.567	**0.746**			
KS	0.297	0.468	0.457	0.283	**0.812**		
KST	0.002	0.303	0.007	0.025	0.327	**0.735**	
KE	0.561	0.213	0.233	0.101	0.313	0.523	**0.798**

*The correlations between latent variables and the diagonal are the AVE’s square root mentioned as off-diagonal values in bold numbers.*

#### Structural Model

The structural model is also known as an “inner model.” In the proposed research model, it exhibits the relationships (paths) between the endogenous variable (EL) and exogenous variable (ES). In the structure model, the β-value shows the relationship between the path of dependent and independent variables and the *R*^2^ value predicts the predictive power. Hence, SEM is used to explore the hypothesized model. This study employed a standard bootstrapping technique to attain the significance of path co-efficient, *p*-values, *R*^2^ value, and *t*-values. Standardized root means square residual (SRMR) was to measure the fitness of the structural model was measured. According to Hair et al. (2014) and Henseler et al. (2016) a value of a good model should have less than 0.08 SRMR value. In consequence, the value for SRMR was 0.053, which was lower than the threshold value.

The existing study executed a standard bootstrapping technique with 5000 bootstrap samples and 390 cases to identify the importance of the path coefficients succeeding Reinartz et al. (2009) and Hair et al. (2014). The significance and relevance of the structural model relationships were determined by relating the *t*-values to the critical *t*-values for significance levels of 0.05 and 0.010 for every path coefficient.

#### Hypothesis Testing

Initially, the result revealed that EL has a significant positive influence on ES (β = 0.867, *t* = 2.712, *p* = 0.004), Hence, H1 was supported. Similarly, EL also showed a significant positive impact on KMPs with (β = 0.888, *t* = 6.955, *p* = 0.000), reveals H2 was supported. Consequently, KMPs reveal a positive relationship on ES (β = 0.797, *t* = 8.745, *p* = 0.000) in H3 also supported and identify significant impact. Correspondingly, KMPs as a mediator between EL and ES (β = 0.707, *t* = 5.713, *p* = 0.000) is supporting the H4. Moreover, the result shows that KE moderates between EL and KMP relationship (β = 0.791, *t* = 2.905, *p* = 0.001). Table 4 shows full estimates of the structural model besides measurements concerned with mediating the variables of KMP and moderating variables of KE.

**TABLE 4:** Finding from SEM (full model).

Hypothesis	SD	Path coefficient	*T*-statistics	*P*-values	Decision
EL → ES	0.086	0.867	2.712	0.004	H1 (+), S
EL → KMPs	0.066	0.888	6.955	0.000	H2 (+), S
KMPs → ES	0.097	0.797	8.745	0.000	H3 (+), S
EL → KMPs → ES	0.066	0.707	5.713	0.000	H4 (+), S
EL *KE → KMPs	0.067	0.791	2.905	0.001	H5 (+), S

*Tests of hypotheses are one-tail tests, value of p < 0.05; value of t > 1.96; S, supported; NS, not supported.*

#### Mediating Effect

Hypothesis four stated the mediating relation of KMPs in between EL and ES. According to H1, the total effect (H1) of EL has a significant and positive impact on ES (β = 0.867, *t* = 2.712, *p* = 0.004) (Mentioned in Table 4). However, when KMPs was added as a mediator into the model the total effect reduces and founded not significant (β = 0.160, *t* = 0.905, *p* = 1.018). However, the indirect effect of KMPs was founded significant and positive (β = 0.707, *t* = 5.713, *p* = 0.000). The result shows that the KMPs have a full mediating effect between EL and ES. Variation accounted for (VAF) calculates the enormousness of the indirect consequence in relation to the entire effect (Hair et al., 2012). Hair et al. (2012) specified that mediation conditions for understanding VAF value should follow; no mediation under 0 > VAF < 0.20, partial mediation under 0.20 > VAF < 0.80, and full mediation over 0.80. In the following study, VAF is 82%, which represents the full mediation. Hence, H4 has been accepted and the results of the mediating effect are shown in Table 5.

**TABLE 5:** Mediating effect.

Independent variable	Direct effect	Indirect effect	Total effect	VAF	Hypothesis	Mediation	Decision
Entrepreneurial leadership	0.160	0.707	0.867	82%	H4	Full mediation	Support

#### Moderating Effect

**FIGURE 2:**
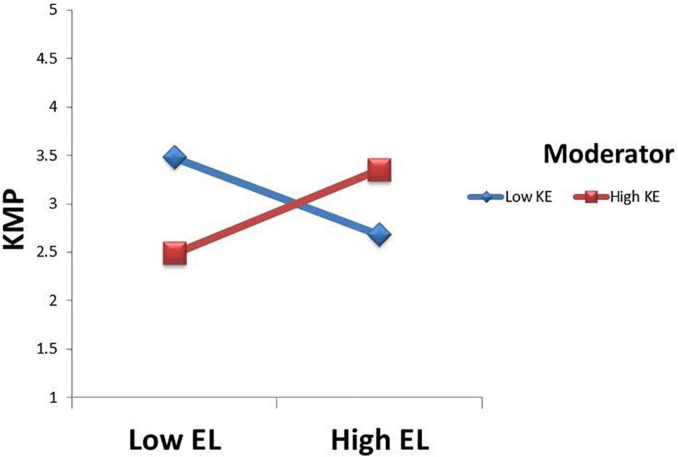
Moderating effect of KE.

This study used the product indicator (PI) approach for understanding the moderation effect of KE. The PI approach is a procedure of estimating latent interactions in structural equation modeling According to H5 moderates KE evaluates the positive relationship between EL and KMPs in the way that the relationship will be stronger when there is high KE (β = 0.791, *t* = 2.905, *p* = 0.001) and supported H5. *F*^2^ value is used to determine the strength of moderating effect. In this study, the value of *F*^2^ was noted as 0.238 with has medium effect size. Moreover, Figure 2 displays the moderating effect, which shows that KE strengthens the relation between EL and KMPs toward the ES.

#### *R*^2^, *Q*^2^, and Effect Size

*R*^2^ value is used to determine the variations in the value of a dependent variable that could be examined by independent variables (one or more than one) (Fassott et al., 2016). The value of *R*^2^ is acceptable according to its demand in the study. Falk and Miller (1992) claim value above 0.10 is acceptable for the *R*^2^; however, Chin et al. (2003) mentioned that the value of *R*^2^ is categorized in three ways, 0.60 is a good value, 0.33 is a moderate value, and 0.19 is a weak value. The obtained *R*^2^ of the current study is 0.16 for ES and 0.357 for KMPs, which shows the 16 and 35.7% variance in the dependent variable. This study follows the Falk and Miller (1992) value of the *R*^2^ statement (see Table 6).

**TABLE 6:** *R*^2^ and *Q*^2^ values.

	*R* ^2^	*R*^2^ adjusted	*Q*^2^ (=1-SSE/SSO)	Effect size
ES	0.16	0.155	0.094	Small
KMP	0.357	0.352	0.165	Medium

The cross-validated redundancy value or *Q*^2^ measures the predictive relevance of the model (Geisser, 1974). It stated that the value of *Q*^2^ is greater than zero so it is considered for predictive relevance. The *Q*^2^ measure size effect has three categories, if greater than 0.000 it is a small effect, if greater the 0.15 it is a medium effect, and if greater than 0.35 it is a large effect (Hair et al., 2014) (Mentioned in Table 6).

## Discussion

The study has given an account of the relationship between EL, KMPs, KE, and ES. The recognition of the proposed hypothesis approves the knowledge-based view theory. Firstly, this study revealed a positive influence of EL toward ES, which suggests that the outcomes concur with (Renko et al., 2015; Al Mamun et al., 2018), which was also the case for the positive association of EL on performance. This shows that leadership style is characterized by pro-activeness, risk-taking ability, innovative thinking, efficient ways of utilizing proper leadership skills, and higher proclivity for ES. In addition, leaders can utilize their entrepreneurial competencies in a competitive environment to achieve ES (Ahmad, 2007; Mitchelmore and Rowley, 2010). This further strengthened our confidence that EL holds a positive significant impact on ES.

Secondly, this study also found a positive significant impact of EL on KMPs. The results endorsed this hypothesis. Leaders are recognized as founders who give foundation to ideologies, vision, and mission (Kuratko, 2007). These foundations help to build consideration of advancement in entrepreneurship. In addition, proper utilization and implementation of knowledge provide support to leadership (Singh, 2008). Therefore, entrepreneurial leaders should provide an open environment for team members to develop solutions, collaborate, and share knowledge (Renko et al., 2015). According to Závodská and Šramová (2018), knowledge contribution and sharing are one of the key factors toward ES. Likewise, it was advised that effective knowledge management implementation entails proactive EL (Chaston, 2012).

Thirdly, this study identifies the significant impact of KMPs on ES. The findings of this study confirm this hypothesis and also confirms the previous findings of KMPs impact on success (Cabrera-Suárez et al., 2001; Chang et al., 2012; Gunasekera and Chong, 2018). The KMPs can substantially increase the chances of ES in tech or IT-based organizations (Chin Wei et al., 2009; Knockaert et al., 2011). Hence, KBV emphasis has remained crucial in explaining the role of KMPs (Grant, 2015). This proved the validity of a theory positing that organizations can achieve superior results through the effective management of knowledge resources (Grant, 1996).

Fourthly, further analysis shows that the indirect influence of EL on ES through the mediation of KMPs has a significant and positive impact. EL through KMP as a mediator increases the level of success in entrepreneurship (Chin Wei et al., 2009; Alshanty and Emeagwali, 2019). This study has confirmed previous research on the mediating role of KMPs (Huang and Li, 2009; Birasnav et al., 2013; Donate and Sánchez de Pablo, 2015; Sadeghi and Rad, 2018). In light of KBV, knowledge is associated with the nature of the job performed by the people in charge and the organizational structure play a pivotal role (Grant, 1996).

Finally, the findings of this study also provide an understanding of the moderating effect of KE which strengthens the relationships between EL and KMPs. Entrepreneurial learning and knowledge give support to businesses for long term survival (Sullivan, 2000). According to Coulson-Thomas (2003), KE helps to improve performance. This shows that knowledge could facilitate success.

### Implications

This present study contributes theoretically to existing literature. It provides evidence that EL holds a strong impact on ES; however, with KMPs and KE it increases the chance of success. Therefore, the following study supports KMPs and KE under the light of KBV theory. Thus, KMPs as mediators and KE as moderators strengthen the relationship between EL to achieve ES. Additional, this study provides new insights, that it is more important for new incumbents (Entrepreneurs) to practice EL skills with KM processes to ensure their safe journey toward ES.

This study has some practical implications for high-tech industry practitioners, small medium enterprises, incubation centers, and researchers in the field of entrepreneurship. Firstly, as stated above this study contributes to literature on EL, KMPs, KE, and ES. Consequently, for better understanding governing and non-governing bodies can derive from this research result. Hence, the graph of not successful ventures could be reduced by introducing KMPs and KE in an EV. Secondly, this study recommended that IT and software owners with knowledge transfer in and out of their organization can develop a strong environment for accomplishing entrepreneurial goals-against entrants.

In managerial implication, mentors, and trainers can help entrepreneurs to polish their leadership skills. In addition, they can mentor entrepreneurs about the operational activities of the venture could be more efficient under KMPs to compete with competitors and aims to achieve ES. Thus, mentors or trainers can highlight the importance of leadership style with accurate knowledge in a specific domain that can help individuals to achieve their desired success. It is noteworthy that success is not only associated with monetary terms it can be non-monetary as well.

### Research Limitations and Future Research Directions

Finally, a number of potential shortfalls need to be considered. First, the sample of study only focused on entrepreneurs of Pakistan. We recommend that further studies should undertake in the different demographic locations and can also conduct comparative studies. Second, cross sectional data were used in the present study. Future work should concentrate on longitudinal data and panel data for better understanding. Third, a quantitative survey method was employed. It is proposed that the qualitative method can also be used to understand the in-depth phenomena, and to collect data for future research. Fourth, the target population was founders, co-founders, and leaders from tech-oriented ventures. It is recommended that samples from other industries, including manufacturing or trade related industries, should be utilized in future studies. In addition, our study was only focused on the EL style to recognize its impact and significance on ES; however, paternalistic leadership, dictator leadership, or any other style could be used to evaluate leadership style in a better way. KMPs as a mediator can also replace technology management processes and supply chain processes. Moreover, gender or age could be used to moderate the relationship and help to analyze the significant difference of gender on the impact of EL on ES.

## Conclusion

Our work has led us to conclude that in the presence of KMP and KE, EL can enable ES. This paper presented and analyzed an integrated research model that links EL to ES through the mediating role of KMPs and moderating role of KE. This investigation supports literature on leadership and knowledge management by demonstrating that the meaningful use of KMPs can let EL have a noteworthy impact on ES. The result reveals that EL would be more successful when KE is implemented. Thus, venture and ES are somehow related to KMPs and KE. Consequently, leaders, CEOs, and managers should adopt KMPs and KE in their daily routine to practice in their EVs.

## Data Availability Statement

The raw data supporting the conclusions of this article will be made available by the authors, without undue reservation.

## Ethics Statement

The studies involving human participants were reviewed and approved by the Professors Committee, Business School, Zhengzhou University, China. As protection of all participants, all subjects read informed consent before participating in this study and voluntarily made their decision to complete surveys. Written informed consent for participation was not required for this study in accordance with the national legislation and the institutional requirements.

## Author Contributions

NH developed the theoretical model, wrote the manuscript, and did an empirical analysis. BL supervised the whole process and reviewed the manuscript writing. Both authors contributed to the article and approved the submitted version.

## Conflict of Interest

The authors declare that the research was conducted in the absence of any commercial or financial relationships that could be construed as a potential conflict of interest.

## Publisher’s Note

All claims expressed in this article are solely those of the authors and do not necessarily represent those of their affiliated organizations, or those of the publisher, the editors and the reviewers. Any product that may be evaluated in this article, or claim that may be made by its manufacturer, is not guaranteed or endorsed by the publisher.
